# Renal structure in type 2 diabetes: facts and misconceptions

**DOI:** 10.1007/s40620-020-00797-y

**Published:** 2020-07-12

**Authors:** Angelo Di Vincenzo, Silvia Bettini, Lucia Russo, Sara Mazzocut, Michael Mauer, Paola Fioretto

**Affiliations:** 1 Department of Medicine, Clinica Medica 3, University of Padova, Via Giustiniani 2, 35128 Padova, Italy; 2 Department of Pediatrics and Medicine, University of Minnesota School of Medicine, Minneapolis, MN USA

**Keywords:** Diabetic nephropathy, Kidney biopsy, Mesangial expansion, Tubulointerstitial lesions, Morphometric analysis

## Abstract

The clinical manifestations of diabetic nephropathy are similar in type 1 and type 2 diabetes, while the renal lesions may differ. Indeed, diabetic glomerulopathy is the predominant renal lesion in type 1 diabetes, although also tubular, interstitial and arteriolar lesions are present in the advanced stages of renal disease. In contrast, in type 2 diabetes renal lesions are heterogeneous, and a substantial number of type 2 diabetic patients with diabetic kidney disease have mild or absent glomerulopathy with tubulointerstitial and/or arteriolar abnormalities. In addition, a high prevalence of non-diabetic renal diseases, isolated or superimposed on classic diabetic nephropathy lesions have been reported in patients with type 2 diabetes, often reflecting the bias of selecting patients for unusual clinical presentations for renal biopsy. This review focuses on renal structural changes in type 2 diabetes, emphasizing the contribution of research kidney biopsy studies to the understanding of the pathogenesis of DKD and of the structural lesions responsible for the different clinical phenotypes. Also, kidney biopsies could provide relevant information in terms of renal prognosis, and help to understand the different responses to different therapies, especially SGLT2 inhibitors, thus allowing personalized medicine.

## Introduction

Renal disease (hereafter referred to as diabetic kidney disease or DKD) affects approximately 40% of patients with diabetes, and is the most common cause of end-stage renal disease (ESRD) worldwide, accounting for almost half of patients on renal replacement treatment in the USA [1]. In the 2016 European Renal Association (ERA)—European Dialysis and Transplant Association (EDTA) registry annual report, 25% of patients starting renal replacement therapy (RRT) were affected by diabetes [2]. The development of DKD in both type 1 and type 2 diabetes is the main predictor of mortality, and is associated with a worst prognosis in diabetic compared to non-diabetic subjects [3]. DKD is strongly associated with increased cardiovascular risk; indeed a large majority of patients with DKD die of cardiovascular complications before reaching ESRD [4]. The clinical manifestations of DKD are common to those of other chronic kidney diseases, i.e., proteinuria, declining GFR and hypertension, while the lesions underlying renal dysfunction are typical of this disease. This is especially true for type 1 diabetes, where the constellation of thickening of the glomerular basement membrane (GBM), mesangial expansion, mesangial nodule formation, and afferent and efferent glomerular arteriolar hyalinosis are pathognomonic of diabetic nephropathy-DN. As discussed below, not all DKD patients will have the ‘classical lesions of DN, especially true of patients with type 2 diabetes. In type 2 diabetes (T2D), in addition to the typical glomerular lesions, the severity of tubulointerstitial and vascular lesions and global glomerulosclerosis lesions may be out of proportion to the classical glomerular lesions of DN, possibly reflecting additional factors such as obesity, insulin resistance, aging, hypertension, hyperlipidemia and atherosclerosis. Also, non-diabetic renal diseases, isolated or superimposed on classic diabetic nephropathy lesions are commonly found in patients with type 2 diabetes, often reflecting the bias of selecting patients for unusual clinical presentations for renal biopsy [5].

The development of DN is usually observed in patients with long-standing diabetes and is frequently accompanied by other microvascular complications, especially, diabetic retinopathy. The diagnosis is typically made through the association of clinical manifestations and laboratory findings consistent with renal damage. In the last decade, however, it has become evident that there are different clinical phenotypes of diabetic kidney disease expression, especially kidney dysfunction manifesting as low GFR with normal or minimally elevated urinary albumin excretion rate (AER) and the underlying renal pathology and natural history of this clinical presentation is not yet fully understood. Thus, deeper understanding of the histopathological mechanisms of renal damage in diabetes could be helpful in stratifying the risk of progression, and hopefully, in ultimately guiding therapeutic strategies. For example, definition of the histological lesions underlying abnormalities in functional parameters could be particularly important in understanding the response to new drugs such as sodium-glucose cotransporter 2 (SGLT2) inhibitors, which have potent effects in slowing progression towards ESRD in type 2 diabetes [6, 7].

### Renal structural changes in diabetes

The development of microalbuminuria has been considered the first manifestation of DKD, although it is now well established that impaired GFR can occur in presence of normal albuminuria. Renal structural lesions in diabetes develop and progress over many years in clinical silence; by the time microalbuminuria or low GFR occur, abnormalities in kidney histology are well established [8]; thus, early recognition of DN at the initial stage should be promoted, given that histological lesions of growing severity become progressively less susceptible to treatment. However, renal biopsy is not routinely performed in diabetes except in particular clinical settings. The indications for renal biopsy in diabetes clinical practice remain much debated, with criteria varying greatly among the different centers, this accounting for different results [5].

The Renal Pathology Society developed a system of classification of DN renal pathology [9]. This system classified type 1 and type 2 diabetes lesions together. They classified these biopsies into four classes or categories: glomerular basement membrane thickening (class I), mesangial expansion (class IIa, mild, and class IIb, severe), until the formation of Kimmestiel-Wilson nodules (class III). The class IV corresponds to advanced glomerulosclerosis. In this classification glomerular lesions are considered the predominant histologic finding, while tubulointerstitial and vascular lesions are considered as complementary additions to clinical prognosis. It is important to consider that this classification was based on renal biopsies performed for clinical indications, presumably in patients with fast progression or other atypical clinical presentations of DN and, consequently, does not provide information on renal structure for the majority of patients with diabetes [10]. Also, DN in type 1 and type 2 diabetes was considered as if it was a uniform disease, which is often not the case [11].

Despite its widespread use, this classification could often be misleading in type 2 diabetes, where the degree of proteinuria and renal dysfunction may be independent of the severity of ‘classical’ diabetic glomerulopathy, and where severe vascular and tubulointerstitial lesions could be the predominant picture, this without the presence of definable non-diabetic renal disease [10, 11]. Finally, histological features of DN somehow differ between type 1 and type 2 diabetes [12].

Indeed, although the clinical manifestations of DN are similar in type 1 and type 2 diabetes, the histological lesions and their progression may differ in the two conditions. In particular, in type 1 diabetes, glomerular injury is always present, with worsening of glomerulopathy in the advanced stages, when the tubuleointerstitial and vascular lesions are usually also present. In contrast, many patients with type 2 diabetes developing microalbuminuria or overt proteinuria may show minimal or no glomerular lesions [11, 12].

### Type 1 diabetes

Glomerular basement membrane (GBM) thickening represents the earliest renal lesion in type 1 diabetes, detectable as early as 2 years after the onset of diabetes [13]. Measurable mesangial expansion usually manifests later and does not correlate as closely with diabetes duration as does GBM width [14, 15]. However, these alterations are always present at the advanced stages of DN, and are responsible for albuminuria/proteinuria and for the reduction of filtration surface of glomerulus and of GFR [16]. These lesions are consequent to the accumulation of specific extracellular basement membrane matrix components including type IV collagens, laminin and fibronectin. In the advanced stages of mesangial expansion (diabetic glomerulosclerosis), Kimmestiel-Wilson nodules are frequently observed. These are nodular lesions resulting from marked mesangial matrix accumulation, forming large round mesangial zones compressing glomerular capillaries. Although more typically present in advanced DN, they may be present early in the evolution of DN, for example, in normoalbuminuric type 1 diabetic children with otherwise mild DN changes and may therefore not always represent more advanced (class III) DN injury as defined in the Renal Pathology Society classification. Arteriolar hyalinosis is an exudative lesion, whereby plasma components, such as immunoglobulins, fibrinogen, complement components and albumin, replace vascular smooth cells. Arteriolar hyalinosis involving both afferent and efferent arterioles is virtually diagnostic of DN and usually develops after few years of diabetes [7, 17]. Abnormalities of the glomerular-tubular junction can be observed once proteinuria develops. These abnormalities are regularly observed in proteinuric patients with DN, often in conjunction with GFR loss, while they are rare in microalbuminuric or normoalbuminuric patients [18]. Tubules may be normal or atrophic, or, in proteinuric type 1 diabetic patients, atubular glomeruli can be observed in > 15% of glomeruli when no tubular connection is identifiable while a similar fraction of glomeruli may have complete obstruction of the glomerular-tubular junction [18]. These abnormalities may vary in development and progression among patients with type 1 diabetes, and even higher heterogeneity can be observed in type 2 diabetes.

Although GBM thickening and mesangial expansion are strongly correlated, in type 1 diabetes some patients have GBM thickening out of proportion with mesangial expansion or vice-versa [14] while tubular-interstitial abnormalities are observed mainly in advanced disease [19] and may contribute to the progression to ESRD. Data from cohort studies evaluating renal biopsies from type 1 diabetic subjects have shown a great variability in the severity of glomerular lesions among the different stages of the disease: in particular, while all patients with microalbuminuria showed abnormal glomerular structure, some normoalbuminuric subjects had glomerular lesions similar to those of microalbuminuric patients [20, 21]. Furthermore, a particular subset of patients with normoalbuminuria showed advanced glomerulopathy with a concomitant reduction of GFR [21]. Despite the variability in the severity of glomerular lesions, overall, in type 1 diabetes AER correlates significantly with GBM width and mesangial expansion, while the most important driver of GFR loss is mesangial expansion [14].

### Type 2 diabetes

In type 2 diabetes the situation is more complex; indeed, heterogeneity of renal lesions has been observed in both microalbuminuric (MA) and proteinuric (P) patients [11], with only a minority of patients showing the typical pattern of DN, while in most of diabetic subjects mild or even absent glomerular lesions were present, with different levels of tubulointerstitial and arteriolar abnormalities. Thus, based on histologic findings, we have described three different categories of structural changes in type 2 diabetic patients undergoing research kidney biopsies [11]:

- I: normal or near-normal renal structure; 35% of microalbuminuric patients and 10% of proteinuric patients can be included in this category.
- II: typical diabetic nephropathology, characterized predominantly by diabetic glomerulopathy. This pattern is present in 30% of patients with microalbuminuria and 50% of those with proteinuria.
- III: atypical pattern of renal lesions; this pattern is characterized by relatively mild glomerular involvement and disproportionately severe tubulointerstitial and/or vascular lesions (arteriolar hyalinosis and atherosclerotic lesions and global glomerulosclerosis).

It is not clear if this variability in patterns of injury in type 2 diabetes is due to concomitant factors, such as arterial hypertension, atherosclerosis or aging, or if the heterogeneity in renal lesions reflects the heretgeneous pathophysiology of type 2 diabetes. In this regard, it is interesting to note that patients in category II (typical glomerular lesions) usually have a long-lasting history of diabetes, a worst metabolic control and they all have diabetic retinopathy, 50% proliferative and 50% background; in contrast, only half patients in both category I and III have retinopathy, all background. Thus, similar pathophysiological mechanisms, responsible for both retinal and glomerular lesions, could explain the development of the renal lesions characteristic of category II.

Moriya et al. recently confirmed the heterogeneity of histological lesions in Japanese patients with type 2 diabetes, suggesting that the presence of typical glomerulopathy at baseline predicts a faster decline of GFR over time, without correlation with the degree of albuminuria [22].

As far as structural–functional relationships in type 2 diabetes, based on electron microscopic morphometric analysis, the data are scarce; an old study performed in Japan demonstrated correlations between glomerular structure and renal function similar to those observed in type 1 diabetes [23], although more recent data suggest a high incidence of normal glomerular structure among MA and P Japanese type 2 diabetic patients [24]. In type 2 Pima Indians with type 2 diabetes, no significant difference in glomerular structure was observed between patients with normoalbuminuria (NA) and those with MA [25], while those with P had more advanced diabetic glomerulopathy lesions. In our cohort of Caucasian type 2 diabetic patients we observed that glomerular structural parameters were, on average, worst going from NA to MA and P (unpublished observations). However, several patients, despite persistent MA or P, had normal glomerular ultrastructure, thus confirming our observation by light microscopy. Moreover, compared to patients with type 1 diabetes and comparable renal functional abnormalities, diabetic glomerulopathy was less advanced in patients with type 2 diabetes. Overall, we found a significant, although imprecise, direct correlation between albumin excretion rate and both GBM width and Vv(Mes/glom), while GFR was inversely related to Vv(Mes/glom) but not to GBM width. We compared the relationships between albumin excretion rate (AER) and morphometric measures of glomerular structure in type 1 and type 2 diabetic patients by cluster analysis and found that around 1/3 of type 2 diabetics fall outside the cluster of structural–functional relationships containing the type 1 diabetic patients [26]. This is due to the fact that a subset of type 2 diabetic patients has increased AER despite the paucity of diabetic glomerulopathy lesions. These findings further strengthen our initial description by light microscopy of heterogeneity in renal structure among patients with type 2 diabetes [11]. Finally, we have reported that GFR loss was significantly correlated with the degree of diabetic glomerulopathy lesions [GBM width and Vv(Mes/glom)] in a large cohort of type 2 diabetic patients during a 4 years of follow-up [27].

A follow-up study with repeated kidney biopsies in Japanese type 2 diabetic patients with and without microalbuminuria reported a correlation between mesangial matrix deposition and GFR loss, while there was no correlation with albuminuria [28]. A similar study performed in Pima Indians with, on average, normoalbuminuria and elevated GFR, showed no correlation between glomerular lesions and very modest GFR decline, while there was a correlation with albumin-to-creatinine ratio [29]. However, in a Pima Indian cohort with type 2 diabetes with higher baseline levels of albuminuria, 46% of whom developed renal function loss ≥ 40%, classical DN glomerulopathy lesions, especially mesangial expansion, were strongly predictive of this progression [30].

Thus, the nature of abnormal albuminuria in type 2 diabetes is complex and, in addition to the classic lesions of diabetic glomerulopathy, other lesions or processes of podocyte or tubular function.

A reduction in the number/density of podocytes has been described in Pima Indians with type 2 diabetes and P [25]. In this population Meyer et al. reported that a lower number of podocytes per glomerulus at baseline was the strongest predictor of the increases in AER al 4 years of follow-up [31]. These observations suggest that podocyte loss is important in the progression to overt nephropathy, rather than in its early development. In our cohort of type 2 diabetic patients [32], we described that the density of podocytes per glomerulus [Nv(epi/glom)] was lower in MA and P than in NA patients. Also, the absolute number of podocytes per glomerulus (Epi N/glom) was lower in MA and P patients compared to controls; in addition, MA and P patients had increased foot process width (FPW) compared to NA patients. AER was significantly correlated with all these parameters. GFR was weakly related only to the density of podocytes. Several patients with abnormal AER had normal Vv(Mes/glom) (≤ 0.25); thus we compared their podocyte structure to that of normoalbuminurics with normal Vv(Mes/glom) [32]. Patients with abnormal AER had lower Nv(epi/glom) and higher FPW than NA patients. Similar results were observed in a similar study of Caucasian type 2 diabetic patients [33]. Overall, these data suggest that in type 2 diabetic patients podocyte loss and changes in podocyte structure occur from the early stages of diabetic nephropathy and might help to understand the nature of albuminuria, especially in those without the classic diabetic glomerular lesions. However, more recent studies in Pima Indian research kidney biopsy subjects with type 2 diabetes found no predictive value of podocyte numerical density on early changes in albumin excretion or measured GFR nor was podocyte number per glomerulus predictive of 40% or more GFR loss [29, 30]. Clearly, more work is needed on this important cell in DN.

### Non-diabetic renal diseases in patients with type 2 diabetes

Non-diabetic renal diseases (NDRD) can be observed in type 2 diabetes, alone or in association with typical diabetic glomerulopathy, further affecting the prognosis and the management of diabetic subjects. In fact, the literature is suggesting that, in type 2 diabetes, NDRD is present with a prevalence higher than expected, and the correct diagnosis through renal biopsy is fundamental to avoid delaying in treatment of these “unanticipated” disorders. Once again, this is based on clinical renal biopsies which are biased towards unusual clinical presentations, while in research biopsies in Caucasian, Japanese, and Pima Indian cohorts, NDRD incidence is low. The real prevalence of NDRD in subjects with diabetes is not precisely defined, ranging from 30% to less than 10% in the different studies. This is probably due, as noted above, to the variability in the indication of renal biopsy and to the fact that kidney biopsies are rarely performed in patients with diabetes. Centers with more liberal biopsy criteria find NDRD far less often than centers with more restrictive criteria [5]. However, this represents a relevant point in clinical practice, because the possible overlap of diabetic renal disease with NDRD, or the misdiagnosis of DN could wrongly guide therapeutic approaches. A recent meta-analysis, including 4876 patients from 48 studies, found a prevalence of 36.9% (ranging from 3 to 82.9%) for isolated forms of NDRD, and a prevalence of 19.7% (ranging from 4 to 45.5%) for NDRD superimposed to DN [34]. The most frequent NDRDs were IgA nephropathy, membranous nephropathy and focal segmental glomerulosclerosis. A recent retrospective study of clinical renal biopsies performed on 1604 Chinese diabetic patients, almost all type 2 diabetes, showed that 44.7% of patients presented lesions diagnostic for DN, while 49.1% had isolated NDRD, and only 6.2% had NDRD associated with DN lesions [35]. Among patients with NDRD, the majority had membranous nephropathy, followed by IgA nephropathy. Only 61of these 1604 patients had type 1 diabetes, and among them non-diabetic nephropathy was observed in 49.2% of cases.

Again, these high rates of NDRD observed among these several studies is probably markedly overestimated because, in most cases, the biopsy was performed because of a high clinical suspicion of NDRD; in fact, the true prevalence is likely less than 10%. So, a comprehensive definition for the indication of renal biopsy in clinical practice should be proposed to correctly distinguish patients with diabetic or non-diabetic renal disease. The diagnosis of DN is usually clinical and applied to patients with long-standing diabetes, concomitant albuminuria, eGFR reduction, hypertension and other microvascular complications, especially advanced retinopathy, in the absence of a high level of suspicion of other conditions. Clinical and laboratory manifestations of DN resemble those observed in many other renal diseases, and kidney biopsy is usually performed in presence of an atypical course, like rapidly developing nephrotic range proteinuria, fast GFR decline, short diabetes duration and absence of diabetic retinopathy. This represents a relevant bias for the real prevalence of NDRD, which is likely to be lower than estimated [36].

### Clinical relevance of renal biopsy studies in type 2 diabetes and future perspectives

As mentioned above, kidney biopsies are rarely performed in diabetic patients with clinical renal findings, based on the assumption that the renal dysfunction is consequent to DN. In addition to the importance of kidney biopsies for the diagnosis and treatment of NDRD, we believe that kidney biopsy is a useful diagnostic tool in patients with type 2 diabetes for the following reasons: (1) renal prognosis, (2) understanding of the different clinical phenotypes (3) understanding of the different responses to different therapies, thus allowing personalized medicine (4) benefit of reassurance to many type 2 diabetic patients with microalbuminuria that they have minimal DN lesions and thus, are at low risk of substantial loss of GFR in near future; the value to patients of such reassurance is often vastly underestimated. In this regard, the heterogeneity of renal lesions has an important influence on progression of DKD and is likely to be responsible for the different clinical presentations in type 2 diabetes. Patients in category II are prone to a faster GFR decline compared to those with mild or absent glomerular lesions. This has been demonstrated in a large number of patients, where the severity of diabetic glomerular lesions was significantly associated with GFR loss [27, 30]. In contrast, patients with mild or no glomerular lesions have slower GFR decline and are probably more likely to revert from microalbuminuria to normoalbuminuria, or to remain stable over time and not progress.

As mentioned above, the classic natural history of DN, with microalbuminuria as first clinical manifestation of renal dysfunction progressing to overt proteinuria and GFR loss, is changing, with a large number of patients with type 2 diabetes presenting with decreased GFR and normoalbuminuria. The UKPDS reported that, during a 15 years follow-up of newly diagnosed patients 14% developed low GFR, 25% became micro or macroalbuminuric and 14% had both [37]. In a large Italian study, among 28,344 patients, 11% had isolated low GFR, 24% micro or macroalbuminuria, and 12% had both. Interestingly, among patients with low GFR, 52% were normoalbuminuric [38]. This emerging phenotype of DN could, in part, be consequent to the widespread use of renin-angiotensin system blockers, which are known to suppress albuminuria and may mask albuminuria progression. The use of new glucose-lowering agents with established antiproteinuric effects is likely to increase the prevalence of this phenotype. Unfortunately, the structural lesions underlying the normoalbuminuric renal impairment phenotype remain largely unknown. Only one small study investigated renal histology in patients with type 2 diabetes, low GFR, and different levels of albuminuria (from normo to macroalbuminuria) and described heterogeneity in renal lesions, ranging from mild lesions to classic glomerulopathy to tubulo-interstitial fibrosis [39]. This contrasts with findings in type 1 diabetes, where patients with low GFR and NA (more often women) have more severe diabetic glomerulopathy than patients with normal GFR [40].

Finally, studies of renal structure could be fundamental to the understanding of the different responses to treatments of individual patients. Recently two glucose-lowering classes of drugs have been shown to have nephroprotective effects. In particular SGLT2 inhibitors were consistently demonstrated to reduce albuminuria and, importantly, GFR loss and ESRD. Cardiovascular outcome trials have shown that this class of drugs significantly reduce major adverse cardiovascular events, hospitalization for heart failure, and renal events [41–43]. The nephroprotective effects have been confirmed in the CREDENCE trial, performed in patients with type 2 diabetes and advanced DN [5]. It is important to point out that the anti-albuminuric response to SGLT2 inhibition markedly varies among patients, and it is reproducible upon re‐exposure [44, 45]; indeed, only 65% of patients had a reduction in albuminuria of at least 30%. The variability in the antialbuminuric response supports the concept that there are responders and non-responders to SGLT2 inhibitors in terms of nephroprotection [44, 45]. It could be of great importance to understand if the nephroprotective effect occurs in patients with classic diabetic glomerulopathy or if the best responders are those with tubulointerstitial lesions. Also, to understand the mechanisms responsible for the nephroprotective effects of this class kidney biopsies could be fundamental and helpful in the discovery of new and more targeted nephroprotective agents.

Given the spectacular effects in RCT, it is tempting to hypothesize that SGLT2 inhibitors could lead to reversal of DN lesions (glomerular? tubulointerstitial?); this important question can be addressed only with longitudinal kidney biopsy studies. So far, only long-term normoglycemia, obtained with pancreas transplantation, has been shown to be able to reverse the established lesions of diabetic glomerulopathy in patients with type 1 diabetes; this ‘proof of concept’ study demonstrated that reversal of diabetic renal lesions can occur in humans [46]. However, the long delay in DN healing, not present at 5 years [47], but demonstrable after 10 years of normoglycemia [46] makes it unlikely that rapid DN glomerulopathy reversal largely accounts for the clinical benefits of the SGLT2 inhibitors; it is more likely that these agents influence the progression of tubulointerstitial injury.
